# Beyond the Alpha Subunit: Pharmacological Modulation of Kv4.2 Channels by Ancillary Proteins

**DOI:** 10.3390/cells15070628

**Published:** 2026-03-31

**Authors:** Kathya Villatoro-Gomez, Rocío Gabriela Sanchez-Olivares, Tania Ferrer

**Affiliations:** 1 Departamento de Ingeniería Química y Bioquímica, Instituto Tecnológico de Colima—Tecnológico Nacional de México, Av. Tecnológico #1 Col. Liberación, Villa de Álvarez 28976, Mexico; kathya.villatoro@colima.tecnm.mx; 2 Centro Universitario de Investigaciones Biomédicas, Universidad de Colima, Av. 25 de Julio 965 Col. Villas San Sebastián, Colima 28045, Mexico

**Keywords:** Kv4.2, KChIP, DPP, ancillary subunits

## Abstract

**Highlights:**

**What are the main findings?**

- The pharmacology of Kv4.2 channels is not an intrinsic property of the pore-forming subunit but emerges from its association with accessory subunits such as KChIPs and DPP6/10.
- Accessory subunits critically shape the pharmacological profile of Kv4.2, so the same compound can display different potency or efficacy depending on the specific subunit composition present in the heteromeric complex.

**What are the implications of the main finding?**

- Because the pharmacological properties of Kv4.2 are determined by tissue-specific accessory subunits, drug responses are expected to vary across organs such as the heart and brain.
- Targeting the Kv4.2 macromolecular complex rather than the pore-forming subunit alone may help guide the development of more selective pharmacological strategies for disorders involving A-type potassium currents, including conditions affecting cardiac and neuronal excitability.

**Abstract:**

Kv4.2 channels are the principal mediators of the fast transient outward K^+^ current (I_tof_) in the heart and the A-type current (I_A_) in the nervous system, both of which play a relevant role in shaping cardiac action potentials and neuronal excitability. This review focuses on how interactions with ancillary subunits, such as potassium channel interacting proteins (KChIPs) and dipeptidyl peptidase-like proteins (DPPs), beyond regulating trafficking, membrane expression, and gating properties of Kv4.2 channels, significantly influence channel drug response, demonstrating that Kv4.2 does not represent a fixed pharmacological entity but rather a dynamic macromolecular complex whose drug responsiveness depends on its subunit composition. Understanding this accessory subunit-dependent modulation is important, as the pharmacological profile of Kv4.2-containing channels may differ depending on the predominant accessory subunit composition in each tissue.

## 1. Introduction

Potassium channels are a large family of transmembrane proteins, widely distributed across organisms, that are involved in different key cellular functions. In the human body, they play an important role in the activity of excitable cells, particularly in the nervous and cardiovascular systems.

There are four major types of potassium channels: calcium-activated, inwardly rectifying, voltage-gated, and two-pore domain potassium channels [1], which are mostly composed of four subunits. These subunits can be identical (homotetramers) or closely related (heterotetramers), and each of them has a distinctive structural domain that is responsible for potassium selectivity (pore-loop). An exception is the two-pore domain potassium channels, which are formed by two subunits, each with two pores, and together form the functional channel [1,2].

The voltage-gated potassium channel family (Kv) is the largest group of ion channels, comprising 40 distinct Kv channels that are divided into 12 subfamilies. These channels regulate cellular excitability and also contribute to processes such as migration, proliferation, and cell volume regulation [3]. Within these channels, there are four closely related subfamilies: Kv1 (Shaker), Kv2 (Shab), Kv3 (Shaw), and Kv4 (Shal), where Kv1–Kv3 mediate a broad range of electrophysiological phenotypes such as fast and slow inactivating outward K^+^ currents, while Shal/Kv4 channels primarily mediate fast A-type K^+^ currents [4,5,6,7,8,9,10].

The Kv4 family can be divided into three subfamilies: Kv4.1 (KCND1), Kv4.2 (KCND2), and Kv4.3 (KCND3) [11], which are found in different tissues, being abundantly expressed in the heart and brain. While each of these isoforms has important functions, this review focuses on the pharmacological modulation of the Kv4.2 channel by ancillary subunits. This channel plays a crucial role in forming the fast transient outward K^+^ current (I_tof_), which is essential for regulating cardiac action potential repolarization, and the A-type current (I_A_), which modulates dendritic integration and synaptic plasticity in specific brain regions [12,13].

## 2. Kv4.2 Channel: Structure, Localization, and Functional Roles

**Figure 1:**
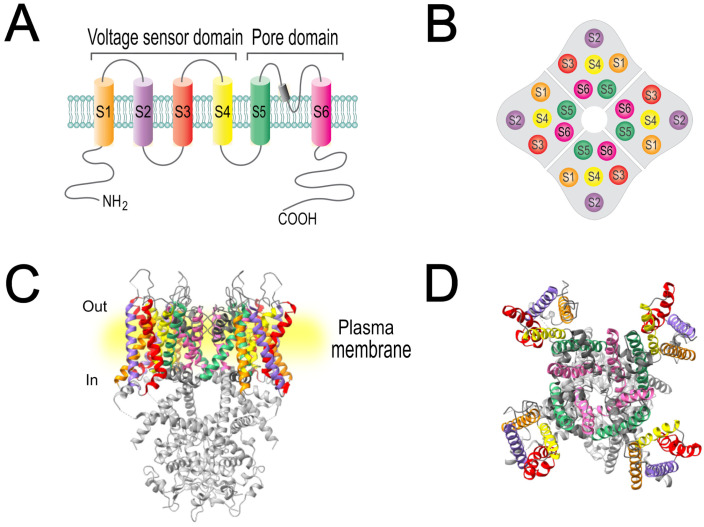
Schematic representation of the Kv4.2 architecture. (**A**) Membrane topology of a single Kv4.2 subunit, indicating the voltage-sensing domain (S1–S4) and the pore-forming region (S5–S6). (**B**) Tetrameric organization of the Kv4.2 subunits forming the functional channel around a central pore. (**C**) Crystal structure of the tetrameric assembly of the Kv4.2 channel (accession code 7F0J) in front view. (**D**) Top view of the same structure. In panels (**B**–**D**), the transmembrane segments are displayed using the same color code defined in (**A**). All three-dimensional structures shown throughout the manuscript were rendered using ChimeraX (San Francisco, CA, USA; v. 1.10.dev202501180816).

Structurally, Kv4.2 consists of four identical pore-forming α-subunits, each containing six transmembrane segments (S1–S6), with the N-terminal (amino acids 1–39) and C-terminal (amino acids 537–630) regions oriented intracellularly (Figure 1A). Segments S1–S4 constitute the voltage sensor domain, while S5–S6 form the pore region (Figure 1A). The complete protein has dimensions of approximately 75 Å × 75 Å × 100 Å. In addition, they have an N-terminal tetramerization domain, known as T1, which mediates their assembly into a tetrameric complex (Figure 1B–D), thereby ensuring proper channel formation [12].

Because Kv4.2 channels play a crucial role in regulating cardiac and neuronal excitability, understanding their tissue distribution is essential to comprehend their physiological role. Although most studies have focused on nervous and cardiovascular systems, Kv4.2 has also been detected in other tissues, highlighting the importance of studying Kv4.2 channels in many other physiological functions.

### 2.1. Kv4.2 in the Central Nervous System

The presence of the Kv4.2 channel in the central nervous system has been identified in regions such as the CA1 region and dentate gyrus of the hippocampus [14,15,16]; cerebellum, specifically in Purkinje and granule cells [16,17]; thalamus, in the medial geniculate body [16,18], and cortex, although this shows relatively lower expression levels [14].

In 1997, a landmark study by Hoffman et al. [19] showed that transient I_A_ in the dendrites of CA1 pyramidal neurons prevents rapid and long-lasting dendritic depolarizations, regulating action potential backpropagation [19].

This finding was later confirmed using a combination of electrophysiological and imaging techniques, in which Kv4.2 was identified as the major molecular correlate of this current in hippocampal CA1 neurons, placing these channels as a key piece in the regulation of dendritic excitability, calcium-dependent signaling, and synaptic plasticity [20].

In 2006, the presence of the channel was confirmed by electrophysiological studies that evaluated the somatodendritic I_A_ in pyramidal neurons of the CA1 region of the hippocampus, highlighting the preferential localization of Kv4.2 in dendritic spines [21]. In addition, the excitatory postsynaptic distribution of Kv4.2 suggests a critical role in modulating synaptic excitability and signal integration [14]. Its presence in presynaptic structures as mossy fiber terminals indicates that Kv4.2 may also have additional yet undescribed functions [17].

### 2.2. Kv4.2 in Cardiac Tissue

Until approximately 25 years ago, the presence and function of the Kv4.2 channel in the human heart had not been fully recognized. In 1996, the Kv4.3 channel was identified as the main component of the I_tof_ current, first in the canine heart and later also suggesting its presence and function in rats and humans. Although the Kv4.2 channel was mentioned, its contribution was only discussed in rats [22].

Subsequent studies based on molecular biology techniques such as PCR and Northern blot in human cardiac tissues demonstrated the presence of the Kv4.2 channel [23]. Although these studies focused on Kv4.3, they suggested that Kv4.2 also plays a crucial role despite its lower expression levels. Its heterogeneous distribution may allow coordinated work between both channels, contributing to the adequate functioning of cardiac contraction [22,24]. Several authors have reported that the channels associated with this current are not expressed homogeneously in cardiac tissue. These channels are more abundant in the epicardium than in the endocardium, contributing to transmural differences in action potential shape and repolarization times across the heart [25,26,27,28,29,30,31].

Despite the importance of the articles mentioned above in establishing that Kv4.2 is a fundamental component of the early repolarization of the cardiac action potential, studies in humans have been limited. The available information comes mainly from works that highlight the role of the Kv4 family and their co-expression with accessory proteins [12]. The only article that describes the function and participation of Kv4.2 channels in humans reports their involvement in nocturnal atrial fibrillation, where a point mutation in the KCND2 gene that alters the kinetic properties of the Kv4.2 channel was shown to play a fundamental role in I_tof_ [32].

At the cellular level, Kv4.2 channels are also distributed heterogeneously. By using techniques such as immunofluorescence and immunoelectron microscopy in rats, it was determined that in atrial cells, they are found in greater proportion in the peripheral sarcolemma and intercalated disks and in lesser proportion in transverse tubules, the latter being very scarce in these cells [33]. However, the Kv4.2 channel was also identified throughout the entire cell membrane, with the highest intensity signal detected in the transverse-axial tubular system rather than in the peripheral sarcolemma [33]. This distribution and its widespread presence in the cell membrane, where they form molecular complexes with Kv4.3 channels and accessory subunits, allow for adequate transport of K^+^ ions during the early repolarization phase and have relevant physiological implications [34].

### 2.3. Kv4.2 in Other Tissues

Despite its recognized expression in the aforementioned systems, there are few but compelling reports of Kv4.2 presence in other organs and tissues, suggesting a broader physiological role than previously thought. Kv4.2 has been identified in smooth muscle, and there are reports of its expression in vascular [32,35] and intestinal tissue [36], in which its function appears to be mediated by I_A_ and plays an important role in the excitability and contractility of these tissues. For example, in 2023, its presence was detected for the first time in the human umbilical vein, where it seems to be an important part of maintaining adequate fetal oxygenation [37]. In intestinal tissue, I_A_ was first characterized in murine colonic myocytes, which led to an investigation where it was found that, despite the fact that there is a greater expression of the Kv4.3 channel, the Kv4.2 channel is also present in these types of cells [36,38].

Furthermore, in 2006, this channel was characterized for the first time in ovarian granulosa cells, where it was described as a possible target for sex steroid hormones. Kv4.2 channel mRNA and protein were also identified in human endocrine cells and in rhesus macaque ovary cells, confirming its physiological importance in this system [39].

Although there are few studies addressing the localization of the Kv4.2 channel in other tissues, the altered expression of this channel has been reported in various types of cancer, such as gastric [40], lung [41], and breast [42] cancer. Among these malignancies, several studies have associated Kv4.2 expression with poor prognosis and adverse clinical features [40,41,43,44]. A possible mechanism proposed to explain this is that Kv4.2 activity promotes the infiltration of M2 macrophages through activation of NF-κB, ultimately accelerating the progression of the disease [40]. Finally, in glioblastoma multiforme, Kv4.2 has been detected in oligodendrocyte progenitor (OPC)-like cells at the cancer–neuron interface, suggesting that this channel plays an important role in the electrical communication of cancer cells and could be a potential therapeutic target in this pathology [45].

## 3. Ancillary Subunits Modulate the Surface Expression and Biophysical Properties of Kv4.2 Channels

Auxiliary subunits act as regulatory components of ion channels, as they co-assemble with the pore-forming α-subunits to alter surface distribution, ion conductance, and channel gating properties. The main ancillary subunits that modulate Kv4.2 channels are Kv channel-interacting proteins (KChIPs) [46] and dipeptidyl peptidase-like proteins (DPPs) [47,48], although other auxiliary proteins can also modify their expression or functional behavior.

### 3.1. Kv Channel-Interacting Proteins

**Figure 2:**
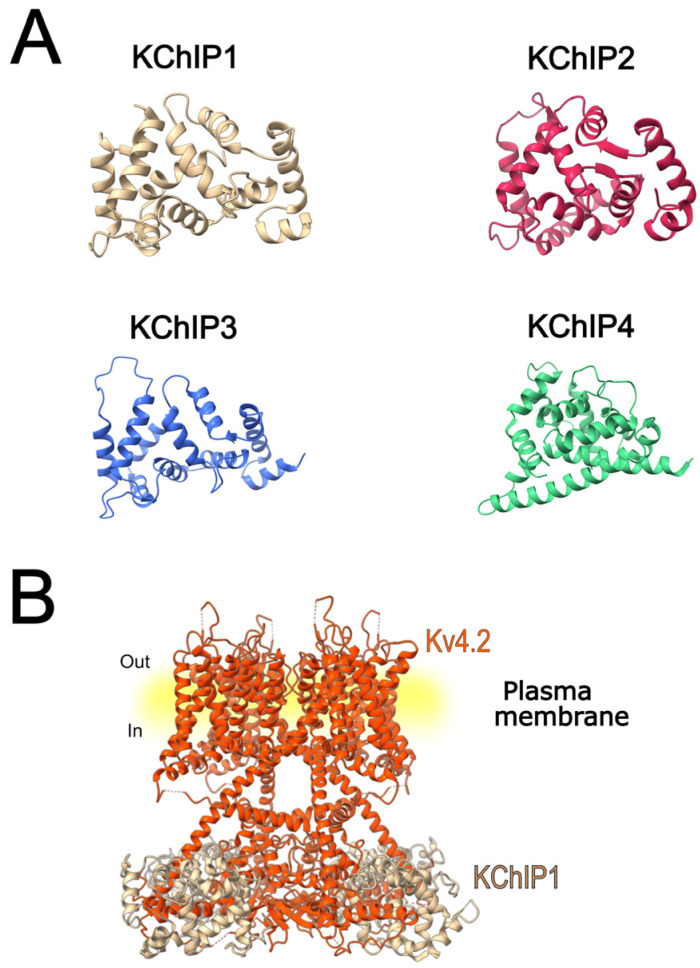
Three-dimensional structures of KChIP proteins and their association with Kv4.2 channels. (**A**) Crystal structures of the four KChIP isoforms illustrating differences in their overall fold: KChIP1 (PDB ID: 1S1E), KChIP2 (PDB ID: 7UKH), KChIP3 (PDB ID: 2JUL), and KChIP4 (PDB ID: 3DD4). (**B**) Three-dimensional structure of the octameric complex formed by the tetrameric Kv4.2 channel associated with four KChIP1 subunits (PDB ID: 7E84), showing the stoichiometric binding of four auxiliary KChIP1 proteins with the cytoplasmic domains of each Kv4.2 channel-forming subunit.

KChIPs are cytosolic proteins encoded by four genes: KCNIP1 (KChIP1), KCNIP2 (KChIP2), KCNIP3 (KChIP3), and KCNIP4 (KChIP4). These genes share a homologous set of seven C-terminal exons that encode the four EF-hands motifs of the core domain. The three-dimensional structure of KChIPs (Figure 2A) reveals that they have N- and C-terminal lobules, each with two EF-hand domains, which surround a deep hydrophobic pocket that mediates their interaction with the cytoplasmic amino-terminal region of Kv4 α-subunits (Figure 2B) [49]. According to Jerng and Pfaffinger 2014 [50], only EF-3 and EF-4 of the C-terminal lobe bind Ca^2+^; while in the N-terminal lobule, EF-1 does not bind divalent cations and EF-2 binds Mg^2+^ [50]. The ability of these ancillary subunits to bind Ca^2+^ suggests that they may regulate cellular excitability in response to intracellular calcium fluctuations [46].

KChIP1 acts as a Ca^2+^ sensor and is predominantly expressed in GABAergic synapses of the hippocampus and cortex. It has been implicated in the regulation of K^+^ channels during GABAergic transmission, enhancing the density of I_A_ and regulating inhibitory excitability [15,51]. Five isoforms of this accessory subunit have been identified: KChIP1a [46], KChIP1b [52], KChIP1-IaΔII, and two other unnamed isoforms [49]. Van Hoorick et al. [52] reported a different expression pattern for each of these variants, detecting KChIP1a in brain, kidney, lung, pancreas, leukocytes, and prostate; in contrast, KChIP1b was found in brain, kidney, liver, placenta, skeletal muscle, small intestine, and testis [52].

KChIP2 is another accessory subunit that has been primarily studied in cardiac tissue and, although to a lesser extent, it has also been examined in the nervous system. It has been shown that by binding to the N-terminal end of Kv4.2 channels, key aspects of their function are modulated, such as density, kinetics, and localization, leading to the correct functioning of the I_A_ and I_tof_ [46]. This subunit is found in the highest proportion in the heart, where it is expressed in ventricular and atrial myocytes and is essential for the regulation of I_tof_. However, its presence has also been detected in certain regions of the hippocampus and cerebral cortex [31,53]. In total, seven transcript variants have been reported for this gene, giving rise to seven distinct isoforms: KChIP2a, KChIP2b, KChIP2s, KChIP2c, KChIP2.5, along with two additional isoforms that have not yet been formally named [49].

KChIP3, also known as Calsenilin, was originally isolated as a Presenilin (PS)-interacting protein but was later found to share ~99% sequence homology with Downstream Regulatory Element Antagonist Modulator (DREAM), a Ca^2+^-regulated transcriptional repressor. Its co-localization with Kv4.2 channels has been observed in the hippocampus, especially in the dentate gyrus [15,54,55,56]. Three isoforms of KChIP3 have been described: KChIP3.1, KChIP3.2, and KChIP3.x. The KChIP3x isoform (also known as KChIP3b) contains a K^+^-channel inactivation suppressor domain (KISD), a transmembrane segment that can modulate the membrane expression of Kv4 channels and slow their inactivation kinetics [57]. Functional studies have shown that this subunit plays an important role in neuroprotection, synaptic plasticity, learning, and memory through various mechanisms such as dissociation from the cell membrane, translocation to the nucleus, and regulation of gene transcription [58,59]. Unlike the other accessory subunits described, KChIP3 can act as a DREAM, in addition to its regulatory function on Kv4.2 channels [58,59].

Like KChIP3, the accessory subunit KChIP4 presents a KISD, which allows it to act as an inhibitor of Kv4.2 channel trafficking to the membrane, retaining it in the endoplasmic reticulum and thus suppressing current density. It affects inactivation kinetics, and its distribution has been almost entirely restricted to the central nervous system [60,61].

KChIP subunits play a crucial role in modulating the voltage dependence, gating kinetics, and surface expression of Kv4.2 channels. Among them, KChIP1-3 proteins enhance the expression of the Kv4.2 channels on the plasma membrane through their interaction with the N-terminal domain of the channel in the endoplasmic reticulum. This interaction masks the intrinsic ER retention signal and facilitates channel trafficking to the cell surface. As a consequence, Kv4.2 channels are retained at the plasma membrane [62,63,64]. On the other hand, there are different reports about the effect of KChIP4 on Kv4.2 surface expression. Some studies report that KChIP4a inhibits Kv4.2 trafficking by binding to the channel and to other KChIPs, forming a ternary plasma membrane complex [57,64,65]. In contrast, another study indicates that KChIP4 increases Kv4.2 expression [66]. Regarding biophysical properties, KChIP1-3 increase Kv4.2 currents by slowing inactivation [67], accelerating recovery from inactivation, shifting the activation curves to hyperpolarized potentials and the inactivation voltage-dependence to depolarized potentials [46,62]. In addition, KChIP subunits reduce N-type inactivation and speed up closed-state inactivation (CSI) [7,66]. Specifically, KChIP2c accelerates the transition of Kv4.2 channels to the closed-inactivated state but greatly reduces the percentage of channels that reach this state [68]. The mechanism by which KChIP subunits regulate Kv4.2 inactivation has not been completely elucidated, but a recent study shows that KChIP1 interacts with the N-terminal and C-terminal helices of the channel, modulating inactivation kinetics by preventing open-state inactivation [12].

### 3.2. Dipeptidyl Peptidase-like Proteins

**Figure 3:**
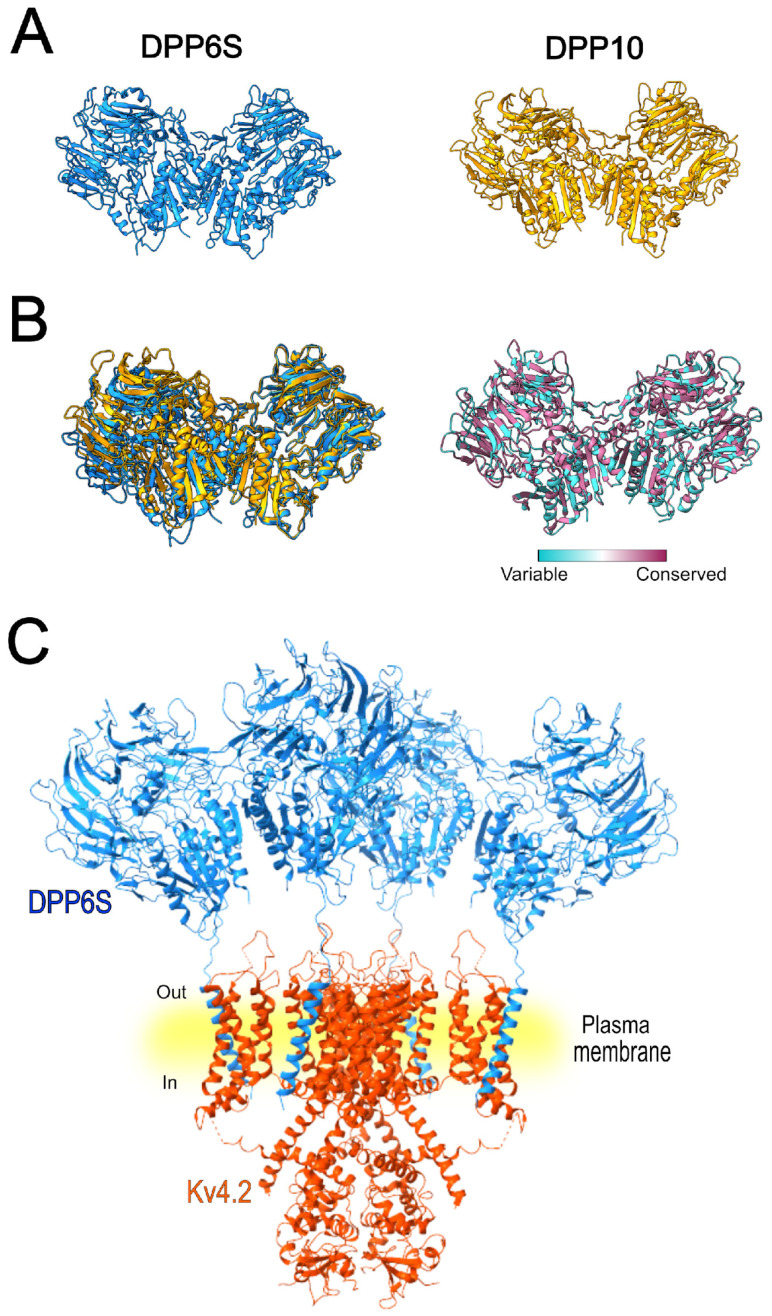
Three-dimensional structures of DPP6S and DPP10 and their association with Kv4.2 channels. (**A**) Crystal structures of the auxiliary subunits DPP6S (PDB ID: 1XFD) and DPP10 (PDB ID: 4WJL) are shown individually to illustrate their three-dimensional architecture. (**B**) Left, structural superposition of DPP6S (blue) and DPP10 (yellow); right, conserved residues mapped onto the structures using a color scale in which conserved positions are shown in purple and variable positions in blue. (**C**) Three-dimensional structure of the Kv4.2 channel in complex with DPP6S (PDB ID: 7E8B), illustrating the association of this auxiliary subunit with the tetrameric Kv4.2 channel.

DPPs are analogous to dipeptidyl peptidases, but they are enzymatically inactive. Their association with Kv4.2 channels has been shown to be necessary for the formation of channels that give rise to I_A_. In 2003, DPPX (later known as DPP6; Figure 3A) was identified as a new component of I_A_, associated with the pore-forming subunit (Figure 3C), and was shown to enhance its trafficking and membrane targeting [47]. Subsequently, DPPY (also termed DPP10, Figure 3A), a related protein that shares structural (Figure 3B) and functional similarities with DPP6 in modulating Kv4 channel complexes, was reported [48,69].

Structurally, both DPP6 and DPP10 are membrane glycoproteins with a short N-terminal domain and a long C-terminal domain [48,69,70,71]. Alternative splicing of the N-terminal region of DPP6 produces two adult isoforms (DPP6-S and DPP6-L) and an embryonic form [70,71,72]. It has been established that the DPP10 gene encodes three different isoforms, distinguished by the inclusion or exclusion of exons that define their N-terminal domain. These isoforms are identified as DPP10a, the long isoform; DPP10c, which has a shorter N-terminal than DPP10a; and DPP10d, the shortest of all and also known as DPP10s [73,74].

The presence of DPP6 has been reported in the brain (cerebellum, hippocampus, cortex, and amygdala), contributing to the modulation of channels in processes such as synaptic excitability and plasticity [50]. It has also been linked to the modulation of Kv channels in the heart, being a fundamental part of the regulation of I_tof_ [75]. On the other hand, using Northern blot analysis, DPP10 was identified in the brain and pancreas, as well as in tissues derived from multiple-sclerosis lesions and retinoblastoma, with only a few transcripts detected in the uterus and colon [48].

DPP subunits have a strong impact on the biophysical properties and subcellular localization of Kv4.2 channels. The co-expression of Kv4.2 with DPP6 or DPP10 increases the current density of Kv4.2 by promoting channel trafficking and facilitating its expression in the plasma membrane [7,76]. These subunits induce a leftward shift in both the conductance-voltage relationship and the steady-state inactivation of Kv4.2 channels and accelerate inactivation and recovery from inactivation [7,47,68,76,77]. Moreover, DPP6 increases the single-channel conductance of Kv4.2 channels [78] and reduces the proportion of channels in the closed-inactivated state [68]. The cryo-electron microscopy structure of the Kv4.2–DPP6S octamer complex was elucidated by Kise et al. [12]. Their findings show that the single transmembrane helix of DPP6S interacts with the S1 and S2 transmembrane helices of Kv4.2, stabilizing the voltage-sensor domain and facilitating the S4 movement. This interaction could underline the faster activation kinetics and recovery from the closed-state inactivation of Kv4.2 co-expressed with DPP6S [12].

### 3.3. Other Accessory Subunits

Although the accessory subunits KChIP and DPP are the most studied in the regulation of Kv4.2 channels, there are other subunits that have been implicated in their modulation, which is consistent with the physiological processes in which they are involved.

KCNE proteins are small transmembrane subunits that modulate the activity of the Kv channel family. They were first identified in 1988, and since then, their association with α-subunits and their involvement in the regulation of these channels have been extensively studied. These auxiliary subunits are small proteins of 100–220 amino acids that each contain a single transmembrane helix [79].

The KCNE gene-encoded protein family consists of five members (KCNE1-5). The first of these, KCNE1, also known as MinK, is primarily expressed in the heart and is co-expressed with Kv7.1 channels [80]. KCNE2 (MiRP1) is also expressed mainly in the heart and is associated with the hERG channel [81]; however, there are reports of its involvement in the regulation of other potassium channels such as Kv7.1 [82], Kv7.2/Kv7.3 [83], and Kv4.2 [84]. KCNE3 (MiRP2) has been found expressed in the epithelium of the colon and stomach, where it is co-expressed with Kv7.1 channels [85], and it has also been detected in cardiac tissue [86]. The KCNE4 (MiRP3) isoform has been detected in non-excitable tissues such as the immune system and stem cells, as well as the heart and brain [86,87]. It has been reported that both KCNE3 and KCNE4 genes undergo alternative splicing to produce short (S) and long (L) isoforms, designated KCNE3S/KCNE3L and KCNE4S/KCNE4L, respectively, which exhibit distinct functional effects. Finally, KCNE5 mRNA has been found in cardiac and skeletal muscle tissues [86,88].

KCNE subunits are also important regulators of Kv4.2 channels. KCNE3S and KCNE3L, coexpressed with Kv4.2 in vitro, decrease Kv4.2 activity by ~30–40% [89,90]. In contrast, KCNE4S barely affects hKv4.2 current density, while it reduces rat Kv4.2 currents by ~40%, and KCNE4L strongly inhibits hKv4.2 currents by 80% at +60 mV [89]. In addition, KCNE4 slows both the activation and inactivation kinetics of Kv4.2, shifts the voltage dependence of activation and inactivation to more positive potentials, and accelerates the recovery from inactivation, producing a significant “overshoot” [91]. This last feature refers to a transient increase in current amplitude above the baseline level, which was also reported in native Ito in human ventricular myocytes [30] and after expression of KCNE2 (MiRP1) with Kv4.2 [84]. Likewise, KCNE2 induces a similar effect, as it also slows both the activation and inactivation kinetics of the channel while shifting the voltage dependence of activation toward more depolarized potentials. Conversely, KCNE3, when co-transfected with Kv4.2 at different ratios in CHO cells, reduces the current amplitude [92].

Although to a lesser extent, other subunits have been implicated in the regulation of the Kv4.2 channel complex. For example, Navβ1, which is encoded by the SCN1B gene, is a subunit considered multifunctional, as it acts as a cell-adhesion molecule and a modulator of Nav channel cell surface expression, kinetics, and voltage-dependence [93]. Similarly, the Kvβ1 subunits are encoded by the KCNAB1 gene, which generates splice variants that produce Kvβ1 proteins with different N-terminal sequences containing between 70 and 90 amino acids (Kvβ1.1–1.3). This variable region is followed by a highly conserved core domain of approximately 330 amino acids, which is characteristic of all Kvβ subunits [94]. Its presence has been identified in the rat brain, particularly in the thalamus [95].

Navβ1 and Kvβ1.2 are additional auxiliary subunits that co-assemble with the Kv4.2 channel, modulating their function. Navβ1 enhances Kv4.2 current density by increasing channel cell-surface expression, although it does not affect the kinetic or voltage-gating properties of the channel [96]. In contrast, Kvβ1.2 does not alter either the current density or the inactivation rate of Kv4.2 but enables the channel to respond to low pO_2_ [97].

Kv channel-associated protein (KchAP) also modulates Kv4.2 channels mainly by enhancing their surface expression through the SUMOylation pathway. This process increases the recycling of Kv4.2 channels to the plasma membrane, augmenting I_A_ and thereby contributing to the regulation of neuronal excitability [98]. However, the effects of KChAP on the biophysical properties of Kv4.2 have not yet been elucidated. Northern blot analysis has shown that this subunit is expressed in different tissues, including heart and brain, with particularly high levels in lung and kidney [99].

Finally, leucine-rich glioma-inactivated protein 1 (LGI1) is a secreted protein that has been studied and detected in the nervous system [100] that contributes to Kv4.2 channel localization through interactions with ADAM22/23. LGI1 promotes Kv4.2 insertion into the plasma membrane, enhancing I_A_ and regulating excitability. In fact, the disruption of LGI1 has been associated with seizure susceptibility [101].

## 4. Assembly of Multiple Ancillary Subunits with Kv4.2 and Their Functional Impact

Currently, several studies have investigated the modulation of the Kv4.2 channel within complexes where multiple accessory subunits interact to determine how one ancillary subunit influences channel behavior in the presence of another.

**Figure 4:**
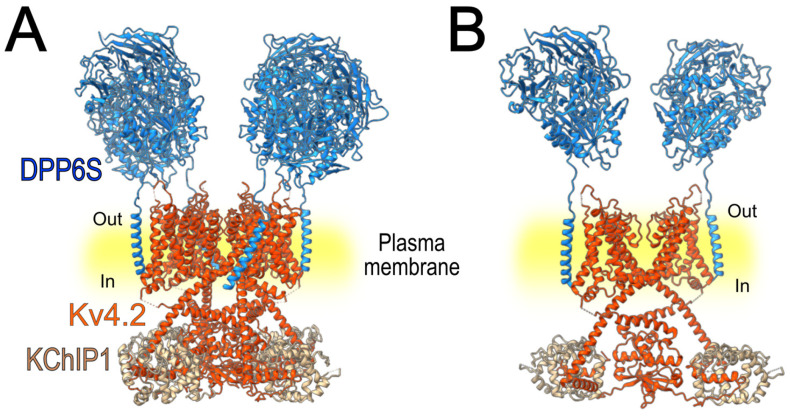
Structural organization of the Kv4.2–KChIP1–DPP6S macromolecular complex. (**A**) Three-dimensional structure of the full heteromultimeric complex composed of the tetrameric Kv4.2 channel associated with four KChIP1 and four DPP6S auxiliary subunits, forming a 12-subunit assembly (PDB ID: 7E8H). (**B**) Simplified representation of the same complex showing two Kv4.2 α-subunits together with two KChIP1 and two DPP6S subunits to facilitate visualization of the spatial arrangement and interactions among the channel and its auxiliary subunits.

A study conducted in 2008 [102] evaluated the modulation of Kv4.2 by DPP6S (DPPX-S), KChIP1, and their combined co-expression with the pore-forming subunit (Figure 4). The currents recorded from the ternary complex exhibited properties like I_A_ in various neuronal populations. While KChIP1 slowed the rate of inactivation and accelerated recovery from inactivation, co-expression of both accessory subunits produced an intermediate effect, slowing inactivation to a lesser extent than KChIP1 alone, and accelerating recovery from inactivation similar to that observed with DPP6 alone [102].

Further supporting these findings, Fineberg et al. [103] reported that co-expression of Kv4.2 with KChIP1 and DPP10a accelerated inactivation, an effect primarily attributed to DPP10a [50,103]. Consistently, Jerng and Pfaffinger [50] expanded on this research by analyzing the modulation of Kv4.2 by KChIP3a and DPP10a, both individually and in combination. In the oligomeric complex, inactivation was slightly slower than with DPP10a alone, indicating the modulatory role of KChIP3a, although DPP10a was the dominant factor. In addition, replacing DPP10a with the DPP6S isoform in the ternary complex resulted in a marked slowing of inactivation kinetics, though the effect did not reach the level observed with KChIP3a alone. An additional experiment, also performed by Jerng and co-workers [104], showed that the rate of recovery from inactivation of the Kv4.2 channel was faster when co-expressed with both accessory subunits (KChIP3 and DPP10), compared to that when it was co-expressed with only one. Moreover, the steady-state inactivation of the ternary complex was shifted toward more hyperpolarized potentials, similar to that with DPP10 alone [104].

Other studies have also reported the differential modulation of Kv4.2 by the ancillary subunits in ternary complexes. Specifically, KCNE3L has no effect on hKv4.2 current amplitude when co-expressed with KChIP2, although it reduces hKv4.2 current when expressed alone with the channel [89,90]. In contrast, hKChIP2 does not prevent KCNE4L-mediated current suppression, which was ~95% at +60 mV in the Kv4.2-KChIP2 complex [89]. Additionally, Levy and coworkers (2010) [91] also compared the properties of the current generated by the Kv4.2 channel co-expressed with KChIP2 and those of the Kv4.2/MiRP3/KChIP2 complex. MiRP3 increases current density in the ternary complex compared with Kv4.2/KChIP2 but does not affect current kinetics or the voltage dependence of channel activation or inactivation [91].

## 5. The Role of Auxiliary Subunits in the Pharmacological Modulation of Kv4.2 Channels

As mentioned above, the Kv4.2 channels underlie I_tof_ and I_A_ in the heart and brain, respectively, and represent an important pharmacological target as multiple drugs used to treat diverse disorders have been shown to modulate their activity. Since ancillary subunits regulate the biophysical properties of these channels, it is not surprising that they also influence their pharmacological behavior, a form of modulation that remains relatively underexplored. However, a major gap in the field is the absence of studies comparing drug effects on Kv4.2 expressed alone versus in combination with one or multiple accessory subunits, under equivalent experimental conditions, which limits understanding of their impact on pharmacological responses. In addition, studies performed in native systems do not permit the clear identification of the accessory subunits or modulatory proteins contributing to the drug response, thereby preventing direct comparison with the effects observed in Kv4.2 expressed alone in the context of the present review. In this section, we examine the role of these subunits in determining the response of the channel to different compounds, highlighting how they can modify both potency and channel function.

### 5.1. Kv4.2 Inhibitors

A representative example of subunit-dependent pharmacological modulation is riluzole, an anticonvulsant drug used to treat amyotrophic lateral sclerosis [105] and major depressive disorder [106]. This drug inhibits Kv4.2 with an IC50 ~190 µM, acting on the closed and closed-inactivated states of the channel, and accelerates the rate of inactivation without affecting activation kinetics [107]. However, these effects are significantly modified by the co-assembly of Kv4.2 with DPP6 or KChIP2c. Co-expression of Kv4.2 with DPP6 increases the inhibitory potency of riluzole (IC50 ~74 µM), but the inactivation kinetics remain unaffected, and the development of closed-state inactivation is faster compared to that when Kv4.2 is expressed alone. On the other hand, in the presence of KChIP2c, riluzole inhibits the current with lower potency (IC50 ~278 µM), shifts the activation curve to the left, speeds up inactivation, decelerates recovery from inactivation, and slows the transition to the closed-inactivated state [68] (Table 1).

These effects can be interpreted in the context of the interactions between accessory subunits and specific channel domains. KChIP subunits bind to both the cytoplasmic N-terminal and C-terminal regions of Kv4.2; however, interaction with the N-terminal domain is considered critical for modulating channel inactivation, as KChIP proteins slow this process [12]. Consistent with this, riluzole has been shown to accelerate current inactivation in the presence of KChIP2c, highlighting the impact of these subunits on state-dependent drug action [68]. In contrast, DPP6 interacts with the transmembrane S1–S2 helices of the voltage-sensor domain [12], and its co-expression with Kv4.2 is associated with faster inactivation kinetics [77]. In this case, riluzole does not further modify this parameter in the presence of DPP6 [68]. Although these structural interactions do not by themselves demonstrate the mechanism underlying the effects of riluzole, they support the idea that accessory subunits define distinct gating properties and drug affinity of Kv4.2 channels, as DPP6 sensitizes the channel to riluzole, whereas KChIP2c has the opposite effect.

Compared to the graded modulation of riluzole action by ancillary subunits, the scorpion toxin AmmTX3 shows a more pronounced dependence on these proteins. AmmTX3 belongs to the α-KTX15 toxin family, and it blocks I_A_ in neurons from the CNS with an IC50 ~0.1 µM, without affecting activation voltage dependence, steady-state inactivation, or kinetics [108,109]. However, despite Kv4.2 predominantly underlying I_A_ in the CNS [20,110], when these channels are expressed alone in a heterologous system, AmmTX3 inhibits the current with an IC50 > 1 µM [111], suggesting that auxiliary subunits increase the sensitivity of Kv4.2 to the toxin. Co-expression of Kv4.2 with KChIP1 does not enhance its sensitivity to AmmTX3 [108], but when Kv4.2 channels are co-expressed with DPP6S, the current is blocked with a potency similar to that observed for I_A_ [108], even in the presence of KChIP1, indicating that DPP6S plays a crucial role in mediating the toxin’s effect (Table 1).

At the structural level, it has been reported that the toxin AmmTX3 appears to bind to the extracellular region of the α-subunit [111]. The single transmembrane helix of DPP6S interacts with the lower part of S1 and the upper half of S2 of Kv4.2 [12]. Therefore, DPP6S may induce structural rearrangements in the Kv4.2 channel that favor inhibition by the toxin [108]. In contrast, in the presence of KChIP1, which binds to the channel intracellularly and thus interacts with the pore-forming subunit in a manner distinct from DPP6S [12], it may promote conformational changes that render the channel less sensitive to toxin inhibition. These findings suggest that DPP6S plays a key role in shaping the extracellular architecture of Kv4.2, thereby facilitating toxin binding.

Another example of a Kv4.2 inhibitor whose action is modulated by an ancillary subunit is 4-aminopyridine (4-AP) [84]. 4-AP is a drug used to facilitate nerve conduction in patients with chronic spinal cord injury [112] and to improve walking ability, as well as cognitive and visual function in patients with multiple sclerosis [113]. This compound inhibits Kv4.2 by binding to the inner pore region when the channel is in the closed state, whereas depolarization-induced channel opening or inactivation promotes drug unbinding. In Kv4.2 expressed alone, this unbinding process occurs relatively rapidly, allowing a reversible and state-dependent blockade. However, in the presence of MiRP1, the 4-AP still binds to the channel in the closed state, but its unbinding is slowed. This results from MiRP1-induced changes in channel gating that hinder the conformational transitions of the inner pore region required for drug dissociation. Consequently, 4-AP remains associated with the channel for a longer time in the presence of MiRP1, compared to Kv4.2 expressed alone, producing a more persistent functional blockade and a delayed recovery of channel activity [84].

### 5.2. Dual Pharmacological Effects on Kv4.2 Channels

An alternative mode of modulation is shown by NS5806, a sulfonylurea compound that enhances Ito in canine cardiac myocytes by slowing inactivation but reduces it in ventricular cardiomyocytes from mice and human-induced pluripotent stem cell-derived cardiomyocytes (hiPSC-CMs) [114,115]. Unlike riluzole, AmmTX3, and 4-AP, which inhibit Kv4.2 currents, NS5806 can either potentiate or suppress these currents depending on the co-expressed auxiliary subunits. Kv4.2 (or Kv4.3) peak current is not affected by the compound when the channel is expressed in Xenopus oocytes [114,115], but in the presence of KChIP2, NS5806 increases the current amplitude and slows inactivation. In contrast, when DPP6-L is also co-transfected, NS5806 inhibits the currents and accelerates inactivation kinetics. This is consistent with the behavior of transient outward current (I_to_) observed in native cells. While Western blot data show that KChIP2 levels remain consistent in all three tissues, DPP6-L expression is predominantly expressed in both mouse ventricular myocardium and hiPSC-CMs and is virtually undetectable in canine ventricular myocytes. Additionally, silencing of DPP6 by using siRNA abolished the I_to_ inhibition by NS5806 in hiPSC-CMs, suggesting that this subunit plays a critical role in the inhibitory action of this compound [115] (Table 1).

As mentioned before, KChIP subunits are located intracellularly and interact with the N-terminal domain of Kv4.2 channels [12]. Previous studies have shown that NS5806 increases the current of Kv4.3/KChIP3 [114] and binds to the C-terminus of KChIP3, thereby enhancing its affinity for the N-terminal domain of Kv4.3 [116]. As KChIP2 and KChIP3 have a near-identical binding site for this compound, it has been suggested that the potentiating effect of NS5806 on Kv4.2/KChIP2 may follow the same mechanism [115]. In contrast, when DPP6-L is part of the Kv4.2/KChIP2/DPP6-L ternary complex, NS5806 reduces the current, likely due to a shift in its binding preference within the complex. The N-terminus of DPP6 interacts with the C-terminus of KChIP2, where the NS5806 binding site is located, and this may hinder NS5806 binding to KChIP2 and favor its association with DPP6. This shift in binding may promote conformational changes that switch the effect of NS5806 from potentiation to inhibition [115].

### 5.3. Modulation of Kv4.2 Channel Complexes by Nanomaterials

Another example of differential Kv4.2 current modulation by an ancillary subunit involves multi-walled carbon nanotubes (MWCNTs). These nanocarriers are used to deliver biomolecules and drugs [117,118] and have been extensively studied for cancer therapy [119,120], although they also have toxic effects on cells [121,122,123]. MWCNTs accelerate recovery from inactivation of Kv4.2 channels expressed alone without affecting current decay. However, in the presence of KChIP2, MWCNTs accelerate current inactivation, suggesting that MWCNTs interfere with the ability of KChIP2 to modulate Kv4.2 channel inactivation [121]. In addition, prolonged exposure to MWCNTs reduces Kv4.2 surface expression without affecting that of KChIP2, indicating a possible disruption of the Kv4.2–KChIP2 interaction by uncoupling the channel from its accessory subunit [121] (Table 1).

**Table 1:** Effects of riluzole, AmmTX3, NS5806, and MWCNTs on Kv4.2 alone and in complex with accessory subunits.

Drug	Effects of the Drug on Kv4.2 Alone	Effects of the Drug on Kv4.2 in Combination with Different Accessory Subunits	References
Riluzole 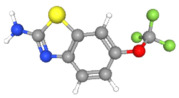	Kv4.2 Inhibition IC50 ~190 µM It acts on the closed and closed-inactivated states ↑ rate of inactivation No changes in the activation kinetics	Kv4.2 + KChIP Shifts the activation curve to the left ↓ inhibition IC50 ~278 µM ↑ Inactivation ↓ Recovery from inactivation ↓ Transition to the closed-inactivated state	[68,107]
		Kv4.2 + DPP6 ↑ inhibition IC50 ~74 µM ↑ development of close-state inactivation No changes in the inactivation kinetics	
AmmTX3 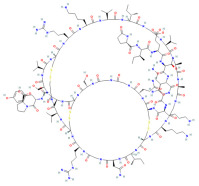	Kv4.2 Inhibition IC50 > 0.1 µM No changes in activation voltage dependence, steady-state inactivation, or kinetics	Kv4.2 + KChIP Shifts the activation curve to the left ↓ inhibition IC50 ~278 µM ↑ Inactivation ↓ Recovery from inactivation ↓ Transition to the closed-inactivated state	[108,111]
		Kv4.2 + DPP6 ↑ inhibition IC50 ~74 µM ↑ development of close-state inactivation No changes in the inactivation kinetics	
4-AP 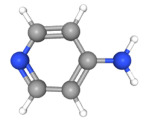	Kv4.2 Inhibition It acts on the closed state Open and inactivated states promote fast drug unbinding.	Kv4.2 + MiRP1 It acts on the closed state ↓ unbinding process Changes channel gating	[84]
NS5806 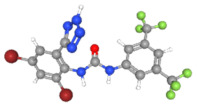	Kv4.2 Minimal or no effect on the Kv4.2 channel alone	Kv4.2 + KChIP ↑ Peak current amplitudes EC50 ~5.3 µM ↓ Current decay ↓ Inactivation	[114,115,124]
		Mouse left ventricular myocytes (Kv4.2/Kv4.3/KChIP2/DPP6-L) ↓ Peak current IC50 ~6.6 µM (total current) IC50 ~12.5 µM (I_tof_)	
		hiPSC-CMs ↓ Peak current IC50 ~8.3 µM	
MWCNTs 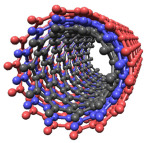	Kv4.2 ↑ Recovery from inactivation No effect on current decay	Kv4.2 + KChIP ↑ Current inactivation ↓ Kv4.2 surface expression without affecting KChIP (↓ Kv4.2–KChIP2 interaction)	[121]

Chemical structures of riluzole, AmmTX3, 4-AP, and NS5806 were obtained from PubChem (NCBI) [125], whereas the multi-walled carbon nanotubes model was generated by the authors. PubChem Compound Identifiers (CIDs): riluzole (CID 5070), AmmTX3 (CID 146018911), 4-AP (CID 1727), NS5806 (CID 11642685). ↑ indicates an increase or acceleration; ↓ indicates a decrease or slowing.

## 6. Therapeutic Implications

The Kv4.2 channel, as previously described, is broadly expressed across the human body; however, its functionally relevant distribution is predominantly restricted to the nervous and cardiovascular systems [12,13], where it constitutes a pharmacological target for different compounds. Studying the effects of drugs on Kv4.2 is essential to understand how individual compounds interact with the channel core, as this provides information about the voltage dependence of the effect, state-specific binding, and gating regulation [107,108,114].

However, Kv4.2 channels assemble into multimeric complexes that include ancillary subunits such as KChIPs and DPP-like proteins, which modify their electrical properties [46,67,126] but may also modulate drug potency and efficacy and even determine whether a compound acts as an inhibitor or activator [68,109,115,127]. Therefore, testing drug effects in experimental models incorporating accessory subunits is essential for predicting how candidate compounds will behave in native tissue [57,66]. In fact, compounds that appear potent in vitro may lose efficacy or switch their mode of action in vivo due to subunit-dependent gating or altered drug binding sites [68,109,115,127].

Although the drugs mentioned in the previous section have been shown to modify the properties of the Kv4.2 channel, both in the absence and in the presence of accessory subunits, more evidence is required to establish how these effects can be translated into specific treatment in neurological or cardiac pathologies.

In the cardiovascular system, Kv4.2 contributes to form I_tof_, and decreased expression or impaired function of Kv4.x channels are associated with electrical remodeling in cardiac hypertrophy and heart failure, leading to reduced I_tof_ and increased susceptibility to arrhythmias [128]. In fact, gene transfer experiments to overexpress Kv4.3, Kv4.2, or KChIP2 have been shown to reverse cardiac hypertrophy in experimental models [129,130]. Therefore, identifying Kv4.2 channel activators could represent a promising therapeutic strategy for cardiac hypertrophy.

In contrast, in the central nervous system, where Kv4.2 underlies I_A_, the increase in this current has been associated with neuronal hyperexcitability, epilepsy, and autism. This is supported by the identification of a gain-of-function mutation (V404M) that enhances closed state inactivation but impairs inactivation after channel opening, ultimately increasing neuronal excitability [131]. Thus, strategies that increase I_tof_ without affecting I_A_ may help to minimize adverse neurological effects.

Finally, the presence of accessory subunits confers to Kv4.2-containing complexes a distinct pharmacological profile in each cell type, creating opportunities to develop more selective, tissue-specific therapeutic approaches. Targeting Kv4.2 complexes formed by auxiliary subunits predominantly expressed in the heart or brain could allow selective modulation of I_tof_ or I_A_ without affecting other tissues where Kv4.2 is also located. However, the translation of these approaches into clinical applications is challenging due to the limited availability of selective modulators, the structural heterogeneity of Kv4 channel complexes, and the limited number evaluating how specific accessory subunit compositions modulate the pharmacological properties of the channel.

## 7. Conclusions

The pharmacology of Kv4.2 should not be considered a fixed property of the pore-forming subunit, but rather a characteristic of a dynamic macromolecular complex. The response of Kv4.2 to pharmacological agents, as well as its membrane expression and functional behavior, is fundamentally determined by its association with accessory subunits, particularly KChIPs and DPP6/10. Thus, the pharmacological profile of Kv4.2 depends on the specific accessory subunits present in the heteromeric complex. If the expression of these subunits is tissue-specific, the pharmacological response of Kv4.2 will also vary between tissues. Consequently, understanding the subunit composition that shapes Kv4.2 pharmacology is essential for developing targeted, organ-specific therapies.

## Data Availability

No new data were created or analyzed in this study.

## Abbreviations

The following abbreviations are used in this manuscript:

Kv	Voltage-gated potassium channel family
I_tof_	Fast transient outward K^+^ current
I_A_	A-type current
KChIPs	Kv channel-interacting proteins
DPPs	Dipeptidyl peptidase-like proteins
DREAM	Downstream Regulatory Element Antagonist Modulator
KISD	K^+^-channel inactivation suppressor domain
CSI	Closed-state inactivation
KChAP	Kv channel-associated protein
LGI 1	Leucine-rich glioma-inactivated protein 1
hiPSC-CMs	Human-induced pluripotent stem cell-derived cardiomyocytes
MWCNTs	Multi-walled carbon nanotubes
